# Durability and Recoverability of Soft Lithographically Patterned Hydrogel Molds for the Formation of Phase Separation Membranes

**DOI:** 10.3390/mi11010108

**Published:** 2020-01-19

**Authors:** Asad Asad, Masoud Rastgar, Hadi Nazaripoor, Mohtada Sadrzadeh, Dan Sameoto

**Affiliations:** Department of Mechanical Engineering, University of Alberta, Edmonton, AB T6G 1H9, Canada; rastgarf@ualberta.ca (M.R.); hadi@ualberta.ca (H.N.); sadrzade@ualberta.ca (M.S.); sameoto@ualberta.ca (D.S.)

**Keywords:** membrane fabrication, soft lithography, hydrogel-facilitated phase separation, hydrogel mold, nanoporous membrane

## Abstract

Hydrogel-facilitated phase separation (HFPS) has recently been applied to make microstructured porous membranes by modified phase separation processes. In HFPS, a soft lithographically patterned hydrogel mold is used as a water content source that initiates the phase separation process in membrane fabrication. However, after each membrane casting, the hydrogel content changes due to the diffusion of organic solvent into the hydrogel from the original membrane solution. The absorption of solvent into the hydrogel mold limits the continuous use of the mold in repeated membrane casts. In this study, we investigated a simple treatment process for hydrogel mold recovery, consisting of warm and cold treatment steps to provide solvent extraction without changing the hydrogel mold integrity. The best recovery result was 96%, which was obtained by placing the hydrogel in a warm water bath (50 °C) for 10 min followed by immersing in a cold bath (23 °C) for 4 min and finally 4 min drying in air. This recovery was attributed to nearly complete solvent extraction without any deformation of the hydrogel structure. The reusability of hydrogel can assist in the development of a continuous membrane fabrication process using HFPS.

## 1. Introduction

Membrane technology is a well-established method for highly selective separation of a wide variety of contaminants from water [1,2,3,4]. Despite many advantages, membrane separation processes are faced with two critical challenges [5]. The first problem is fouling of the membranes with contaminants, which reduces water flux over time. Membrane fouling is defined as the attachment and accumulation of dissolved materials (water contaminants) or suspended particles on the membrane surface and internal pores. The fouling results in a dramatic reduction of the membrane performance with time and reducing its life span [6,7,8]. The second major challenge is the trade-off relationship between permeability and selectivity of membranes [9]. As permeation characteristics of membranes improved, the rejection capability is reduced [10]. To improve membrane performance and lower the membrane fouling, chemical treatments [11,12] or physical modifications [13,14] have commonly been attempted by either coating the membrane surface with hydrophilic/hydrophobic layers or modifying the membrane matrix by blending with additives (such as nanofillers, surfactants, and polymeric additives). However, these approaches suffer from many disadvantages which restrict their extended applications in practice. Leaching of additives out of the polymer matrix and detachment of surface-coated materials even in mild filtration conditions have been widely reported in the literature [15,16,17].

As an alternative to coatings and surface treatments, membrane surface patterning has recently attracted attention [18,19]. Tailoring the topographical surface of a polymer membrane can theoretically increase the effective surface area, which is directly proportional to the permeate flux. Therefore, the trade-off between permeability and selectivity is partially avoided because a higher effective area is fit into a smaller system, and permeation is significantly increased without sacrificing the selectivity [20]. Furthermore, patterns on the membrane surface can create secondary flows that boost the fluid shear at the vicinity of the membrane surface for certain designs leading to a higher antifouling property for some applications [21,22].

Over the last decade, there have been many efforts to develop new techniques to fabricate membranes with micro and nano-sized features [23,24,25,26,27,28,29]. The current methods to fabricate patterned membranes can be classified as either mold-based patterning or direct printing approaches [23,24,25,26]. In mold-based patterning, phase inversion micromolding (PSµM) [30] and thermal embossing micromolding [31], a solid mold is used to produce the features on the membrane surface. More recently, micropatterned anion exchange membranes have been fabricated using 3D printing techniques [32]. Although these methods have succeeded in producing patterned membranes, some drawbacks are encountered. In the PSµM, the polymer solution is cast on the mold and dipped in the coagulation bath to start the phase separation process. However, such an approach results in a pattern replication on the backside of the membrane. The active surface—the face which contacts the non-solvent first—is still unstructured [33]. Despite the progress in using different phase separation methods to modify the PSµM, this method is limited in the membrane types that are compatible with the technology [28]. Thermal embossing compresses the surface of previously manufactured membranes to form surface features but damages the internal structures of the membrane due to the application of high pressure (approximately 15 bar) and temperatures in the fabrication procedure. There is also an inverse relationship between the height of the features and the membrane permeation response such that an increase in the height of patterns results in a lower flux [34]. In contrast to the PSµM and thermal embossing approaches, direct 3D printing of membranes is in its initial stages of development and currently suffers from limited pattern resolution, materials choice and poor scalability [18].

Hydrogels are water-swollen cross-linked polymers that hold and retain high contents of water owing to their 3D network structure [35]. The existence of water in hydrogel plays a crucial role in the overall diffusion of solutes within the gel matrix [36]. The unique properties of hydrogels in terms of solute uptake and release expands its applications in many fields such as drug delivery [37], tissue engineering [38], cell culturing [39], and biomedical applications [40]. Recently, we reported a novel fabrication method for porous patterned membranes that relies on the high water-content of micropatterned hydrogel molds and named the process as “hydrogel-facilitated phase separation (HFPS)” [29]. HFPS successfully replicated different patterns and shapes on the dense membrane surface without affecting the membrane’s surface chemistry. The HFPS-fabricated membranes have an asymmetric structure containing finger-like pores on the bottom and a dense skin layer on top similar to the conventional non-solvent induced phase separation [1,17]. Reusability of the hydrogel mold is of interest to lower the cost and time required for large-scale membrane fabrication. In HFPS process, a thin layer of polymer solution (consisting of polymer and solvent) is cast on a hydrogel mold and due to the high content of water in hydrogel, phase separation starts at the patterned interface. The solvent from the polymer solution exchanges with the non-solvent from the hydrogel mold forming a porous membrane structure. The gradual buildup of the solvent within the hydrogel mold during consecutive membrane fabrication lowers the performance of the mold and membranes cast from it compared to the ideal initial condition. Thus, the phase separation process is slower since the concentration gradients between the non-solvent filled mold and solvent/polymer mixture are smaller, resulting in different membrane performance from each casting. In this study, we investigated the effect of continuous usage of hydrogel mold on the membrane performance over replication numbers. No available studies, to our best knowledge, investigated the reusability of hydrogel molds for membrane applications. Herein, we developed a procedure to extract the diffused solvent inside the hydrogel during the HFPS method to recover the performance of fabricated membrane into its initial state. This study provides an insight into the continuous fabrication of HFPS membranes using hydrogel molds.

## 2. Materials and Methods 

### 2.1. Chemicals 

Polyethersulfone (PES, Baden Aniline and Soda Factory (BASF), Ludwigshafen, Germany, Ultrason E6020p, Mw = 58 kDa), Polyvinylpyrrolidone (PVP, Sigma-Aldrich, St. Louis, MO, USA, Mw = 350 kDa), and *N*,*N*-dimethylacetamide (DMAc, Sigma-Aldrich, St. Louis, MO, USA), were used to prepare polymer casting solutions. Agarose (Sigma-Aldrich, St. Louis, MO, USA, chemical abstracts service (CAS) number: 9012-36-6) was used to make hydrogel solutions. PMMA (McMaster-Carr) was used to develop master molds. Membrane rejection performance was evaluated through filtering of Dextran (Mw = 500 kDa). All materials were used without any modifications.

### 2.2. Preparation of Polymer Solution

PES polymer solution was prepared by mixing 15 wt% PES, 2 wt% PVP and 83 wt% DMAc and then stirred in a beaker overnight at room temperature until the solution reached a homogeneous state. Thereafter, the beaker containing the polymer solution was placed to rest at room temperature for one day and then used for the membrane fabrication.

### 2.3. Preparation of HFPS Membranes

Hydrogel solution was prepared using the microwave method in which 5 wt% Agarose was mixed with distilled water then heated using a microwave until boiling. The solution was cast on a patterned acrylic master mold, with a thickness of 1.6 mm and left for gelation (2–3 min). The hydrogel mold was gently removed from the master mold and placed on a glass plate with the patterns face up. The polymer solution was subsequently hand cast using a Gardco film applicator on top of the hydrogel mold with a gap thickness of 200 µm. The assembly was kept under a fume hood until the membrane was fully formed (approximately 1 min). Then the membrane was gently peeled off the hydrogel mold and placed in a distilled water bath for later use.

### 2.4. Characterization

#### 2.4.1. Membrane Morphology

The cross-sectional images of the membranes were examined using field emission scanning electron microscopy (FE-SEM, Zeiss, Oberkochen, Germany). Membrane samples were dried overnight at room temperature and broken in liquid nitrogen and then coated with a gold layer (~2 nm thickness) using Denton gold sputter to aid in SEM imaging. 

#### 2.4.2. Water Content and Average Pore Size

The average pore size of each membrane was calculated based on water filtration velocity method [14]

(1) $$r_{m}=\sqrt{\frac{\left(2.9-1.75\varepsilon \right)\times 8\gamma \delta Q}{\varepsilon 2.9A\;\times \;\Delta P}}$$

where *r_m_* is the membrane average pore size, *ε* is the membrane porosity, *δ* is the thickness of the membrane, γ is the viscosity of water (8.9 × 10^−4^ Pa s), *Q* is the flow rate of water passing across the membrane (m^3^/s), *A* is the membrane surface area (m^2^), and ∆*P* is the transmembrane pressure being applied (0.28 MPa). The porosity of each membrane was calculated by a gravimetric method following a standard procedure from the literature [29,41]. From each membrane, samples were cut and immersed in distilled water overnight. The wet membrane samples (mwet) were weighed using a digital balance after ensuring there is no excess water on the membrane surface. After that, the membrane samples were dried overnight at 60 °C and were weighed in dry condition (*m_dry_*). The porosity of the membrane ε is found using:

(2) $$\varepsilon =\left(\frac{\left(m_{wet}-m_{dry}\right)/\rho_{w}}{\frac{m_{wet}-m_{dry}}{\rho_{w}}+\frac{m_{dry}}{\rho_{p}}}\right)\times 100$$

where *m_dry_* is the weight of a dry membrane (g), *m_wet_* is the weight of a dry membrane sample (g), and *ρ_w_* and *ρ_p_* are the densities (g/cm^3^) of the water and polymer, respectively.

#### 2.4.3. Filtration Tests

**Figure 1:**
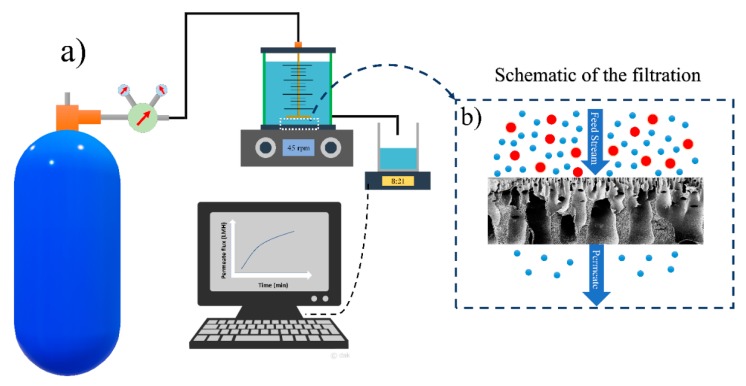
Schematic diagram of (**a**) the dead-end filtration system and (**b**) the filtration mechanism in the membranes.

The water filtration experiments of HFPS membranes were conducted using a dead-end filtration system shown in Figure 1. The surface area of a Millipore cell (Amicon^®^ Stirred Cell 400 mL) was 41.8 cm^2^ and the applied transmembrane pressure was 40 psi. The flux results were calculated using

(3) $$J_{w}=\frac{Q}{A\Delta t}$$

where *J_w_* is the permeated water flux through the membrane (L/(m^2^·h)), *A* is the membrane surface area (m^2^), *Q* is the amount of permeate (L), and ∆*t* is the sampling time intervals.

#### 2.4.4. Solute Rejection

In order to measure the dextran rejection, samples from the permeate and the feed solutions were collected and analyzed using total organic carbon (TOC) instrument (Shimadzu, Model TOC-V; detection range 3–25,000 mg L^−1^, Kyoto, Japan). The rejection and the measured concentrations are related as follows,

(4) $$R=\left(1-\frac{C_{p}}{C_{f}}\right)\times 100$$

where the *C_p_* and *C_f_* is the solute concentration in the permeate and feed samples, respectively.

### 2.5. Treatment Methodologies

The time of treatment required to recover a hydrogel mold is crucial for continuous and large-scale productions. An ideal treatment plan would fully extract the solvent from hydrogel molds without damaging its structure in a short time with minimum energy and material requirements. When a hydrogel mold is used in phase separation, the de-mixing process between membrane polymer solvent (DMAc) and non-solvent (water) changes the hydrogel liquid content. Thus, using the same hydrogel mold for another casting leads to a membrane with different characteristics compared to the initial trials due to the presence of solvent within the mold. The membranes replicated from an untreated hydrogel mold were denoted as M1_pristine, M2_untreated and M3_untreated in which the numbers (1, 2 and 3) represent the order of castings. For the case of untreated hydrogel, the mold was placed in a water bath for 10 min at room temperature and then air dried for four min and used again. To ensure the consistency in the characteristics of the fabricated membranes, the initial state of the mold should be recovered after any castings. Here, two methods are proposed for hydrogel treatment—the first one is based on cold treatment (long term), and the second one involves heat treatment (short term).

#### 2.5.1. Cold Treatment (Long Term)

For long-term treatment, after each membrane casting, the hydrogel mold was flushed with water, and it was placed in a distilled water bath (0.5 L) at room temperature for three days, which allows for natural diffusion of residual DMAc. Before membrane casting, the mold was again flushed with water then gently forced air applied to the hydrogel surface to remove excess water for four min. The membranes produced from a cold treated hydrogel were denoted as M2_cold, M3_cold, M4_cold and M5_cold. 

#### 2.5.2. Heat Treatment (Short Term)

After each membrane casting, the hydrogel mold was placed in a warm distilled water bath (50 °C) for a time between 5 and 10 min and then placed in a room temperature distilled water bath (23 °C) for 4 min. After that, the surface of the mold was air-dried using pressurized air for 4 min. The membranes produced from a heat-treated hydrogel were denoted as M2_hot, M3_hot, and M4_hot. The drying process was included in both treatments to remove the residual water at the surface of the hydrogel mold as it affects the phase separation process. The temperature of the warm water bath was chosen to be 50 °C as its high enough to increase the diffusivity within hydrogel diffusivity while remaining well below the melting point temperature (80 °C) of the agarose to avoid damaging the hydrogel structure.

## 3. Results and Discussion

**Figure 2:**
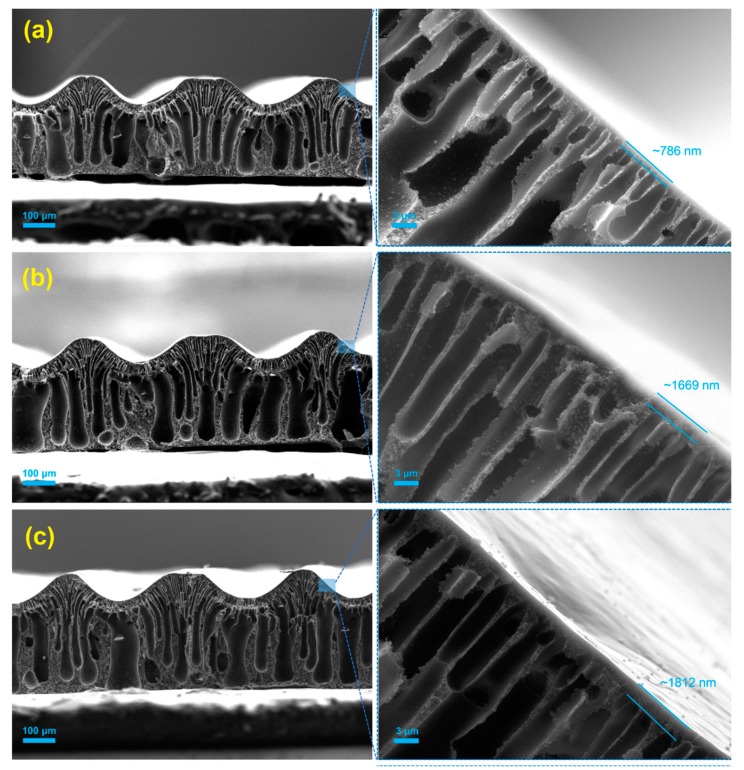
Field emission scanning electron microscopy (FE-SEM) cross-sectional images of (**a**) M1_pristine, (**b**) M2_untreated and (**c**) M3_untreated membranes being fabricated using the same mold without pre-treatment.

The effect of the continuous usage of a hydrogel mold without any treatment on the performance of the fabricated membrane was first characterized by field emission scanning electron microscopy (SEM). SEM images of M1_pristine, M2_untreated and M3_untreated membranes replicated from the same hydrogel mold are shown in Figure 2. All membranes showed a similar asymmetrical finger-like structure which is typical in HFPS-fabricated membranes [29]. The thickness of the top skin layer of the membranes increased from 786 nm in the M1_pristine membrane to 1670 nm in the M2_untreated membrane and 1812 nm in the M3_untreated. This increase in the thickness is attributed to the existence of solvent inside the hydrogel mold which remained from the previous casting.

**Figure 3:**
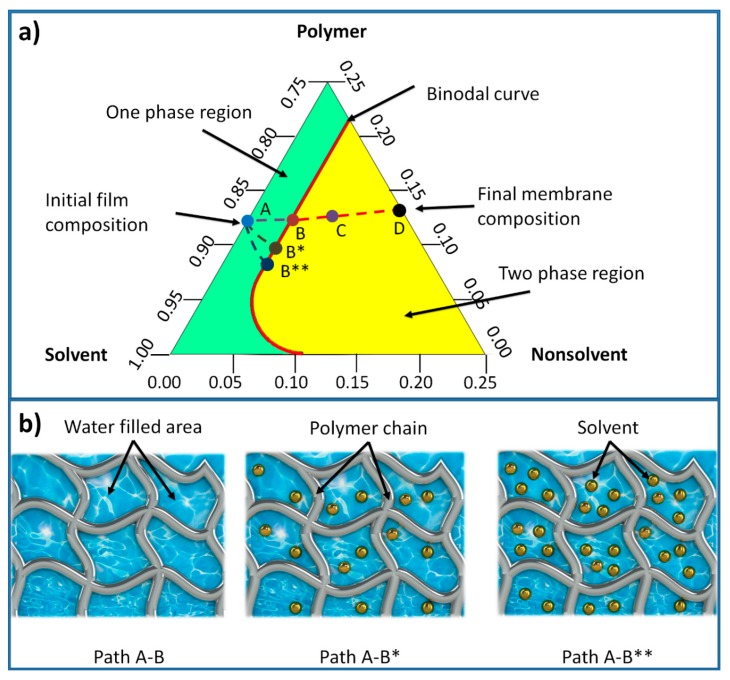
(**a**) Ternary phase diagram of a polymer/solvent/non-solvent system. (**b**) Schematic view of the hydrogel structure with consecutive castings without any treatment. Path A-B shows the time needed for a polymer solution to start precipitation. As the amount of solvent increases in the hydrogel due to the consecutive castings without treatment, the precipitation time becomes longer (Path A-B* and A-B**).

In membrane formation using a non-solvent induced phase separation (NIPS), a three-component ternary diagram of polymer–solvent–non-solvent describes the thermodynamics of the membrane precipitation which is shown in Figure 3 [42]. Each corner of the triangle represents one component such as polymer, solvent, and non-solvent, while any point inside the triangle represents a mixture of these components. The system consists of two distinct regions, separated by a binodal curve: (i) one-phase region where all components are miscible and (ii) two phase-region where the polymer solution separates into polymer-rich that forms the solid part of the membrane, and polymer-lean that forms the pores of the membrane [42]. The entire membrane precipitation process is tracked by the path AD, where point A represents the initial polymer composition and point D represents the final membrane. Point B, on this path, represents the first precipitation of the polymer due to the demixing process between solvent and non-solvent. As the precipitation continues, the concentration of the polymer becomes high enough to be considered as solid material (point C). The last point D on the non-solvent-polymer axis indicates the porosity of the membrane [43]. The existence of solvent in the hydrogel mold increases the precipitation time, as more non-solvent solution (mixture of solvent and non-solvent) is needed to make the polymer solution thermodynamically unstable. Hence, the time required for the system to reach the first precipitation point B (being referred to as path A-B*) increases. With further increase of the solvent content inside the hydrogel, this time increases and subsequently the path A-B** becomes longer [44]. The precipitation time affects the morphological structure of phase separation membranes represented by skin layer thickness and average pore size.

**Figure 4:**
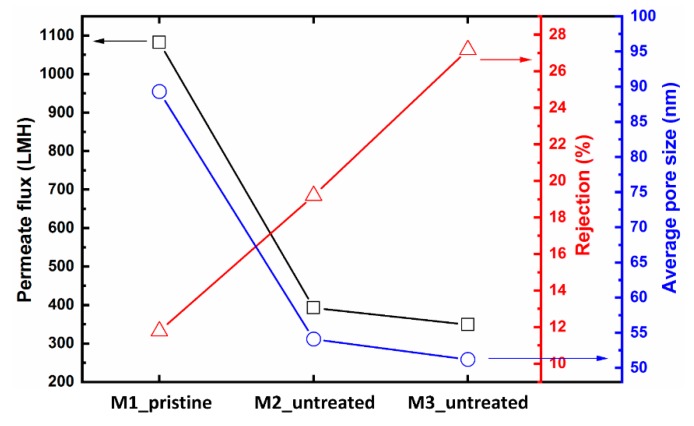
Pure water flux of patterned membranes replicated from the same hydrogel mold. In the first three castings, the mold was just washed with pure water and then reused.

The pore size, pure water flux and rejection results for the M1_pristine, M2_ untreated and M3_ untreated membranes were compared and presented in Figure 4. The average pore size calculations showed a sharp decline from 89 nm (M1_pristine) to 54 nm (M2_ untreated) and gradual decline from 54 nm (M2_ untreated) to 51 nm (M3_ untreated). This decrease in the average pore size is due to the increase of amount of solvent in the hydrogel mold after the first and the second castings. The combined effect of solvent in the hydrogel mold decreased the average pore size and increased the thickness of the skin layer significantly, and dropped the pure water flux of M2_ untreated and M3_ untreated by 63% and 68%, respectively. Moreover, it increased the dextran rejection of M2_ untreated and M3_ untreated by 1.7 and 2.4 times as compared with the pristine membrane (M1_pristine). These results suggest that, if the hydrogel mold is not treated after each membrane casting different membrane performance will be achieved each time.

### 3.1. Cold Treatment

**Figure 5:**
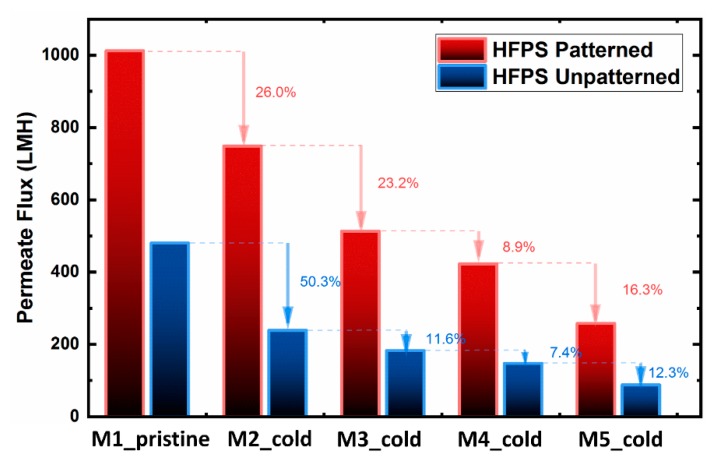
The effect of the cold treatment process on the filtration performance of HFPS patterned and unpatterned membranes prepared from the same hydrogel molds for five castings.

To evaluate the cold treatment protocol, the filtration performance of patterned and unpatterned HFPS membranes was investigated for up to five castings from the same mold (Figure 5). The pure water flux results showed a gradual decline for both patterned and unpatterned membranes over the period of treatments. Although the cold treatment process was used on the hydrogel mold before each membrane casting, the full recovery of the pure water flux was not successful. This decline is attributed to (i) the existence of solvent inside the hydrogel after each casting, and (ii) the permanent deformation of the hydrogel structure. Previous studies on conventional phase separation membranes have shown that the existence of solvent in the coagulation bath decreases the effectiveness of the non-solvent (water) and thus slows down the precipitation rate. The slower solvent/non-solvent exchange rate was found to result in denser structures [44,45]. The permanent deformation of the hydrogel structure also reduces the water-filled areas, thus lowering the demixing rate between the solvent and the non-solvent.

### 3.2. Heat Treatment

Based on the literature, factors that affect the diffusion of solute within hydrogel are the hydrogel structure (gel pore size), properties of solutes (concentration and size), diffusion time and temperature of solutes [46,47]. In most cases, the temperature of solute is increased to a mild temperature, usually between 30–50 °C, which has proven to be an effective range for increasing the diffusion coefficient and therefore a better solutes extraction. Since in our case, the hydrogel recipe and the type of solvent are fixed the other two parameters were considered in the heat treatment process. However, increasing the temperature closer to the melting point of the agarose may damage the hydrogel structure. Therefore, as shown in Table 1, the temperature of the warm water bath was set at 50 °C and the treatment time was varied to evaluate the water flux recovery. Moreover, a cold water bath stage added to lower the temperature and prevent the hydrogel deformation before the drying stage.

**Figure 6:**
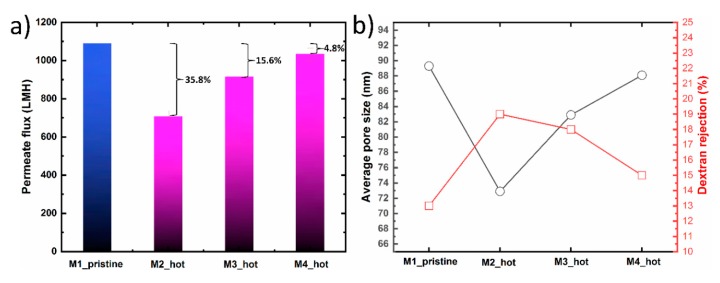
(**a**) Pure water flux of patterned hydrogel-facilitated phase separation (HFPS) membranes fabricated using heat-treated hydrogel mold with an overall filtration time of 400 s for each membrane. (**b**) Average pore size and dextran rejection for the heat-treated membranes.

Figure 6 shows the filtration performance, average pore size and dextran rejection of M1_pristine, M2_hot, M3_hot and M4_hot membranes replicated from the same hydrogel. After the first membrane casting, the hydrogel mold went through three heat treatment process, as shown in Table 1. The filtration performance for M2_hot membrane showed 64.2% water flux recovery as compared to the pristine membrane. This decline in the water flux indicates that the warm water bath time for M2_hot was not enough to extract all DMAc solvent from the hydrogel mold. The existence of solvent in the hydrogel, even if it’s a small amount, has a significant effect on the morphological properties of the prepared membranes. The average pore size and dextran rejection of M2 were 72.9 nm and 19%, respectively. These results show a similar trend compared with M2_ untreated in which no treatment was applied. However, M2_hot is still closer to the M_pristine due to the partial solvent extraction. In the case of M3_hot, the warm water bath time was increased which resulted in higher water flux recovery ~84.4, from one side, and closer average pore size and dextran rejection to the original membrane (M1_pristine). As the warm water bath time increases, the flux recovery percentage increases too, confirming the importance of both time and temperature in solvent extraction. In the fourth membrane (M4_hot), the warm treatment time was 10 min, which resulted in a ~96% flux recovery. This can be attributed to a nearly full solvent extraction without damaging the hydrogel structure. Moreover, the average pore size and dextran rejection were close to those of the M1_pristine. We believe for the M4_hot case, the hydrogel mold state was similar to that of a pristine mold which then results in similarities in water flux, average pore size, and dextran rejection.

## 4. Conclusions

We have demonstrated, for the first time, a simple treatment process that allows repeated usage of the same hydrogel mold in micropatterned phase separation membrane castings. The formation of HFPS membranes relies on the demixing process between solvent from the polymer solution and water contained within the hydrogel mold. The change in the hydrogel mold initial state, significantly affected the membrane formation process and subsequent flux and rejection performance. Our experiments showed that the repeated use of the same hydrogel mold without any treatments resulted in a tighter membrane having smaller average pore size and lower permeated water. Two types of hydrogel mold treatments, cold and heat, proposed in order to extract the diffused solvent from the hydrogel without sacrificing the hydrogel integrity. The proposed plans for improving mold recovery rely on enhancing the diffusion rate of solvent (DMAc) within the hydrogel to increase the degree of solvent extraction from the mold before repeated castings. In the case of the hydrogel cold treatment process, results showed that this method was lengthy and not effective in terms of water flux recovery. Alternatives, the heat treatment process showed a significant improvement in the hydrogel mold recovery represented by water flux recovery in cast membranes. The best heat treatment parameters of those that were tested were found to be 10 min in a warm water bath, followed by 5 min in a cold water bath and 4 min drying time, which resulted in 96% flux performance recovery. It is believed that this combination provided enough time for solvent extraction and relaxation of the hydrogel mold to create similar structure and content to the pristine state. This study provides insight into the advantages and disadvantages of treatment methods that can be used for hydrogel mold recovery in the HFPS method for membrane applications and is the first step in future work to develop optimized recovery protocols for hydrogel molds intended for large-scale production of patterned membrane surfaces. 

## 5. Patents

There is a patent application on the hydrogel-facilitated phase separation (HFPS) method.

Asad Asad; Dan Sameoto; Mohtada Sadrzadeh. Patterned microfilter membrane and method of preparing the same. PCT/CA2018/050838; 10 July 2018.

## Figures and Tables

**Table 1:** Details of heat treatments for recovery of the hydrogel mold in patterned membrane.

Membrane Casting	Warm Water Bath at 50 °C (min)	Cold Water Bath at 23 °C (min)	Drying Time (min)	Average Pore Size (nm)	Dextran Rejection (%)	Water Flux Recovery (%)
M2_hot	5	5	4	72.9	19	64.2
M3_hot	7.5	5	4	82.9	18	84.4
M4_hot	10	5	4	88.1	14	96.2
